# Complete Genome Sequence of *Photobacterium damselae* Subsp. *damselae* Strain SSPD1601 Isolated from Deep-Sea Cage-Cultured *Sebastes schlegelii* with Septic Skin Ulcer

**DOI:** 10.1155/2019/4242653

**Published:** 2019-04-10

**Authors:** Yongxiang Yu, Zheng Zhang, Yingeng Wang, Meijie Liao, Xiaojun Rong, Bin Li, Kai Wang, Jing Chen, Hao Zhang

**Affiliations:** ^1^Key Laboratory of Maricultural Organism Disease Control, Ministry of Agriculture and Rural Affairs, Yellow Sea Fisheries Research Institute, Chinese Academic of Fishery Sciences, Qingdao 266071, China; ^2^Laboratory for Marine Fisheries Science and Food Production Processes, Qingdao National Laboratory for Marine Science and Technology, Qingdao 266237, China

## Abstract

*Photobacterium damselae* subsp. *damselae* (PDD) is a Gram-negative bacterium that can infect a variety of aquatic organisms and humans. Based on an epidemiological investigation conducted over the past 3 years, PDD is one of the most important pathogens causing septic skin ulcer in deep-sea cage-cultured *Sebastes schlegelii* in the Huang-Bohai Sea area and present throughout the year with high abundance. To further understand the pathogenicity of this species, the pathogenic properties and genome of PDD strain SSPD1601 were analyzed. The results revealed that PDD strain SSPD1601 is a rod-shaped cell with a single polar flagellum, and the clinical symptoms were replicated during artificial infection. The SSPD1601 genome consists of two chromosomes and two plasmids, totaling 4,252,294 bp with 3,751 coding sequences (CDSs), 196 tRNA genes, and 47 rRNA genes. Common virulence factors including flagellin, *Fur*, *RstB*, *hcpA*, OMPs, *htpB*-Hsp60, *VasK*, and *vgrG* were found in strain SSPD1601. Furthermore, SSPD1601 is a pPHDD1-negative strain containing the hemolysin gene *hlyA_ch_* and three putative hemolysins (emrA, yoaF, and VPA0226), which are likely responsible for the pathogenicity of SSPD1601. The phylogenetic analysis revealed SSPD1601 to be most closely related to Phdp Wu-1. In addition, the antibiotic resistance phenotype indicated that SSPD1601 was not sensitive to ceftazidime, pipemidic, streptomycin, cefalexin, bacitracin, cefoperazone sodium, acetylspiramycin, clarithromycin, amikacin, gentamycin, kanamycin, oxacillin, ampicillin, and trimethoprim-sulfamethoxazole, but only the bacitracin resistance gene *bacA* was detected based on Antibiotic Resistance Genes Database. These results expand our understanding of PDD, setting the stage for further studies of its pathogenesis and disease prevention.

## 1. Introduction

*Photobacterium damselae* subsp. *damselae* (PDD) is a marine bacterium widely distributed in the marine environment and can infect numerous aquatic animals, including fish, mollusks, crustaceans, and cetaceans [1–3]. PDD was originally isolated from the damsel fish (*Chromis punctipinnis*) in California and described as *Vibrio damsela* [4]. Subsequently, diseases caused by PDD have been reported in wild and cultivable fish in the United States, Spain, Denmark, Japan, Korea, and India, and PDD has gradually become a common pathogen in these areas [2, 5, 6]. In China, the bacterium was demonstrated to be pathogenic in *Chaetodon auriga* and mandarin fish in the 1990s, and the occurrence of disease caused by PDD in aquacultural animals (such as tongue sole, *Epinephelus lanceolatus* L., and *Larimichthys crocea*) has been reported in recent years [7–9]. Furthermore, PDD may also cause human infections, which may lead to necrotizing fasciitis with multiple organ failure and even a fatal outcome [10]. These studies suggest that PDD has gradually become a potential generalist pathogen with the ability to cause disease in a wide range of organisms.

The pathogenic mechanisms and variability in virulence genes of PDD have begun to be elucidated. Previous studies indicated that hemolysins Dly and HlyA_pl_ (renamed phobalysin (PhlyP) owing to its differential characteristics), which are encoded in the plasmid pPHDD1, play a key role in the virulence of PDD strains [11, 12]. The hemolytic activity exhibited by pPHDD1-negative strains was caused by a chromosome-encoded hemolysin termed hlyA_ch_ (PhlyC) and a phospholipase termed PlpV [13]. Recently, the immunogenicity of Hly_ch_ was evaluated, revealing that the virulence might be further enhanced in PDD with the Hly_ch_ gene [14]. Furthermore, it was reported that the CarSR-like system histidine kinase RstB is necessary for regulated production of the three major toxins Dly, PhlyP, and PhlyC [15]. The existence of a notable phenotypic heterogeneity among virulent strains of PDD, together with a wide range of hosts that can be colonized, suggests the existence of other uncharacterized virulence factors. To further understand the pathogenesis and the related virulence factors of PDD, genomic analysis of various PDD strains is crucial.

During the past 3 years, we have investigated the bacterial diversity among deep-sea cage-cultured black rockfish *Sebastes schlegelii* in the Huang-Bohai Sea area in the People's Republic of China. Based on the epidemiological investigation and analysis of bacterial diseases in cage-cultured *S. schlegelii*, PDD is one of the most important pathogens of *S. schlegelii* and is present throughout the year with high abundance in this area [16]. In this study, complete genome analyses were performed to explore the biodiversity and pathogenesis of PDD strain SSPD1601, and the results will provide useful information for the understanding of the potential virulence factors of the pathogen and prevention of PDD infection.

## 2. Materials and Methods

### 2.1. Strain Features and Pathogenic Properties

PDD strain SSPD1601 was initially isolated in Changdao, China, from deep-sea cage-cultured *S. schlegelii* with clinical signs of septic skin ulcer. The pathogenic correlation between SSPD1601 and *S. schlegelii* was determined by intramuscular infection. To obtain a pure culture, a single colony of SSPD1601 was selected and transferred to a fresh trypticase soy broth (TSB) agar plate at least three times. Then, a single colony was selected and incubated in 200 mL liquid TSB medium at 150 rpm and 28°C for 20 h. The morphology of SSPD1601 was assessed by transmission electron microscopy (JEM-1200EX, JEOL, Tokyo, Japan). The strain resources were archived at Yellow Sea Fisheries Research Institute under accession number SSPD1601.

All protocols for experiments involving live animals conducted in this study were approved by the Institutional Animal Care & Use Committee (IACUC), Yellow Sea Fisheries Research Institutes (Qingdao, China), under the approval number IACUC-20170710. We conscientiously abided by the ethical principles of animal welfare and the guidelines of the IACUC.

### 2.2. Genomic DNA Isolation and Whole-Genome Sequencing

The genomic DNA of SSPD1601 was extracted using commercial kits according to the manufacturer's instructions. The quality of the extracted DNA was detected using Qubit (Thermo Fisher Scientific, Waltham, MA, USA) and NanoDrop (Thermo Fisher Scientific) accordingly. Then, the qualified genomic DNA was used to prepare single-molecule real-time (SMRT) bell DNA template libraries with a fragment size >10 kb according to the manufacturer's specification. Genome sequencing was conducted by gene denovo sequencing, and SMRT sequencing was performed on the PacBio RSII sequencer (Pacific Biosciences of California, Inc., Menlo Park, CA, USA) using P4-C2 chemistry. The SMRT raw sequence data were assembled using the hierarchical genome-assembly process (HGAP) pipeline [17]. The overlap layout consensus (OLC) strategy and Quiver consensus algorithm were used to correct and validate the quality of the de novo assembly [17, 18].

### 2.3. Genomic Analysis

GeneMark was used to predict open reading frames (ORFs), while repetitive elements, rRNAs, and tRNAs were identified by RepeatMasker, rRNAmmer, and tRNAscan, respectively [19–22]. The genes were annotated in diverse databases including Gene Ontology (GO), Kyoto Encyclopedia of Genes and Genomes (KEGG), Cluster of Orthologous Groups (COG), nonredundant protein (nr), UniProt/Swiss-Prot, and Pfam. Furthermore, the Virulence Factors of Pathogenic Bacteria (VFDB), Pathogen Host Interactions (PHI), Antibiotic Resistance Genes (ARDB), and Carbohydrate-Active enZYmes (CAZy) were identified through BLAST searches, respectively. Circular genome maps were generated using the Circos (http://circos.ca/) program based on genomic annotation information.

### 2.4. Phylogenetic Analyses

The complete reference genome sequences were downloaded from NCBI and used for comparative genomic analysis based on the average nucleotide identity (ANI) value [23]. The reference strains were PDD strain KC-Na-1 (accession numbers CP021151-CP021156), PDD strain Phdp Wu-1 (CP018297-CP018299), *P. damselae* subsp. *piscicida* strain 91-197 (AP018045-AP018048), *Photobacterium profundum* SS9 (CR354531-CR354532, CR377818), *Photobacterium gaetbulicola* Gung47 (CP005973-CP005974), *Vibrio vulnificus* strain VV2014DJH (CP019320-CP019321), *Vibrio harveyi* strain 345 (CP025537-CP025540), and *Vibrio scophthalmi* strain VS-05 (CP016414-CP016417).

### 2.5. Antibiotic Resistance Analyses

SSPD1601 was resuspended with 15 g/L NaCl solution and adjusted to the concentration of l0^7^ CFU/mL based on the operating instructions of the drug disk manufacturer. The antibiotic resistance phenotype was determined by the Kirby-Bauer disk diffusion test, and the types and concentrations of antibiotics are listed in Table 1. The zones of inhibition were measured after incubation at 28°C for 16 h, and then the antibiotic resistance phenotypes were determined based on the aquatic bacteria susceptibility test standard issued by the American Association for Clinical Laboratory Standardization and subsequently compared with the ARDB analysis.

## 3. Results and Discussion

### 3.1. General Features and Pathogenic Properties of PDD SSPD1601

**Figure 1:**
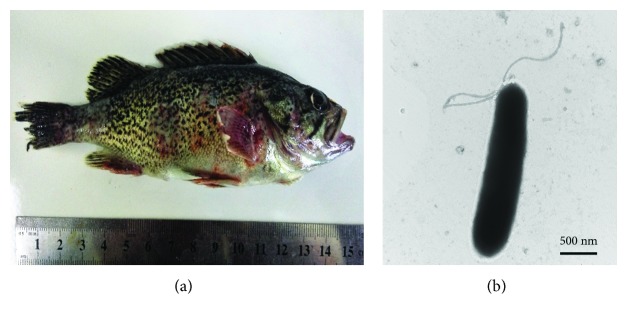
Gross lesions in *S. schlegelii* infected with PDD (a) and electron micrograph of PDD strain SSPD1601 (b).

SSPD1601 was isolated from cage-cultured *S. schlegelii* exhibiting clinical signs of septic skin ulcer. During the early stage of infection, the disease manifested as signs of hemorrhage in the body and the fins, followed by ulcers on the body surface and caudal peduncle [16]. The same clinical symptoms were achieved during artificial infection (Figure 1(a)). Electron microscopy showed that SSPD1601 is a rod-shaped cell approximately 0.5-0.8 *μ*m × 0.8-1.5 *μ*m with a single polar flagellum (Figure 1(b)).

### 3.2. Genomic Properties of PDD Strain SSPD1601

**Figure 2:**
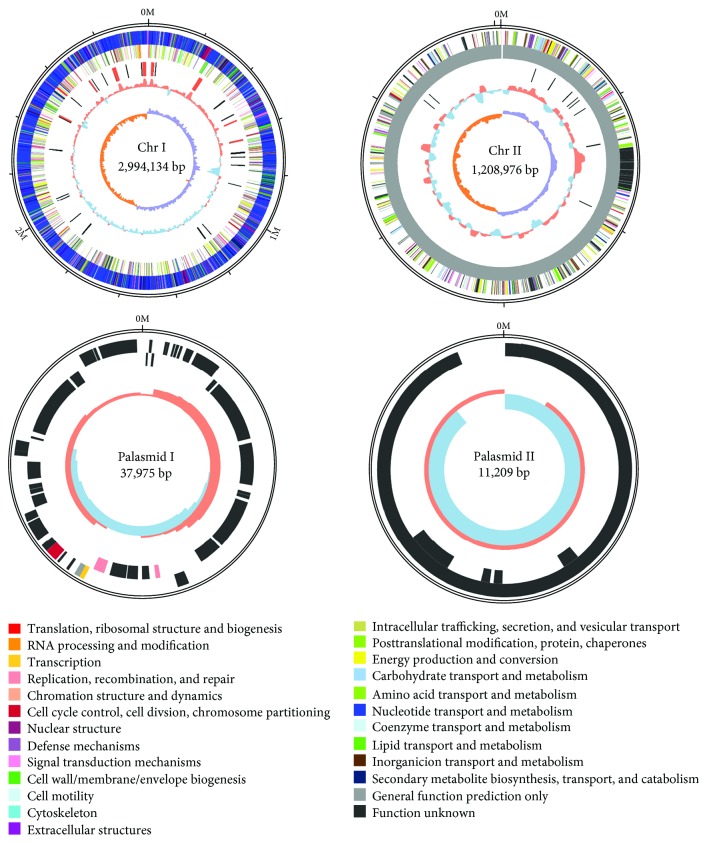
The circular genome maps of SSPD1601. From the outer ring to the inner circle are positive chain genes, negative chain genes, ncRNA (black represents tRNA, and red represents rRNA), GC content (red indicates greater than mean, and blue indicates less than mean), and GC skew (purple means greater than zero, and orange means less than zero).

The complete genome of SSPD1601 comprised a total of 4,252,294 bp, including two circular chromosomes (Chr I, 2,994,134 bp with GC content of 41.37%; Chr II, 1,208,976 bp with GC content of 39.10%) and two circular plasmids (plasmid I, 37,975 bp with GC content of 41.54%; plasmid II, 11,209 bp with GC content of 36.03%) (Figure 2). A total of 2,655 coding sequences (CDSs), 184 tRNA genes, and 47 rRNA genes were detected in Chr I. Chr II contained 1,037 CDSs and 12 tRNA genes. Plasmid 1 consisted of 44 protein-coding genes, and Plasmid 2 consisted of 15 protein-coding genes. The genome sequence data of SSPD1601 have been submitted to the NCBI GenBank database under the SRA accession number PRJNA490082.

### 3.3. Functional Categorization

The results of the COG functional categorization of the predicted ORFs of SSPD1601 indicated that Chr I had a higher number of unigenes than Chr II. The unigenes encoded on Chr I were categorized into 22 COG classes, including categories A (one ORF; RNA processing and modification), B (one ORF; chromatin structure and dynamics), C (182 ORFs; energy production and conversion), D (36 ORFs; cell cycle control, cell division, and chromosome partitioning), E (266 ORFs; amino acid transport and metabolism), F (82 ORFs; nucleotide transport and metabolism), G (137 ORFs; carbohydrate transport and metabolism), H (144 ORFs; coenzyme transport and metabolism), I (74 ORFs; lipid transport and metabolism), J (171 ORFs; translation, ribosomal structure, and biogenesis), K (191 ORFs; transcription), L (139 ORFs; replication, recombination, and repair), M (194 ORFs; cell wall/membrane/envelope biogenesis), N (88 ORFs; cell motility), O (122 ORFs; posttranslational modification, protein turnover, and chaperones), P (170 ORFs; inorganic ion transport and metabolism), Q (42 ORFs; secondary metabolite biosynthesis, transport, and catabolism), R (311 ORFs; general function prediction only), S (232 ORFs; function unknown), T (227 ORFs; signal transduction mechanisms), U (90 ORFs; intracellular trafficking, secretion, and vesicular transport), and V (39 ORFs; defense mechanisms). In contrast, the ORFs on Chr II were categorized into 20 classes (excluding A and B), with 67 ORFs in category C, three ORFs in category D, 95 ORFs in category E, 20 ORFs in category F, 92 ORFs in category G, 26 ORFs in category H, 21 ORFs in category I, 21 ORFs in category J, 90 ORFs in category K, 36 ORFs in category L, 44 ORFs in category M, four ORFs in category N, 47 ORFs in category O, 74 ORFs in category P, 18 ORFs in category Q, 140 ORFs in category R, 90 ORFs in category S, 86 ORFs in category T, 14 ORFs in category U, and 30 ORFs in category V. Furthermore, plasmid I contained five ORFs in categories D, K, L, and R, and plasmid II contained one ORF involved in S only.

### 3.4. Virulence Factors of PDD Strain SSPD1601

Hemolysins are virulence and cytotoxic factors in PDD that have been intensively studied. In this study, PDD strain SSPD1601 showed *β*-hemolysis on sheep blood agar, but the virulence plasmid pPHDD1 was not detected. Furthermore, as a pPHDD1-negative strain, the reported chromosome-encoded *PlpV* and *ColP* genes homologous to hemolysin were not present; only the hemolysin gene *hlyA_ch_* was detected on Chr I. Based on the NCBI databases, the gene sequence of *hlyA_ch_* shares 99% sequence identities with its orthologs in PDD strain RM71. In addition, three putative hemolysins, emrA (sharing 99% coverage and 100% identity with the HlyD family secretion protein of *P. damselae*, WP_106339580.1), yoaF (sharing 94% coverage and 96% identity with the hemolysin of *P. damselae* subsp. *piscicida*, OLQ82859.1), and VPA0226 (sharing 99% coverage and 100% identity with the thermolabile hemolysin of *P. damselae*, WP_106142536.1), were also found to be encoded on the chromosome and may play key roles in the pathogenesis of SSPD1601. Factors responsible for the regulated expression of hemolysins (*RstB* and *hcpA*) were found to be encoded by SSPD1601. Previous studies have indicated that *RstB* and *hcpA* are associated with hemolysin expression in PDD and *V. cholerae* and play a pivotal role in pathogenesis [15, 24]. Additionally, by searching VFDB (http://www.mgc.ac.cn/VFs/main.htm), other potential virulence factors were identified (Supplementary Table S1). Interestingly, a large number of genes (14/50) homologous to genes encoding flagellin (flgC/fliI/flip/flhA; 79-100% sequence identities) and adhesion enhancer (htpB-Hsp60; 79-93% sequence identities) were found to be encoded on the chromosome [25–27]. Another previous study indicated that PDD can infect marine fish and humans through skin wounds or ingestion of infected fish [28]. The discovery of a large number of motility- and adhesion-related genes may help elucidate the invasion mechanism of PDD on the surface of the skin or intestine. In addition, secretion systems in Gram-negative bacteria are associated with exotoxin secretion and cytotoxic effectors and play a pivotal role in pathogenesis [29]. The secretion system-related *VasK* and *vgrG* genes were also found in SSPD1601. On the other hand, *Fur* gene sequences of PDD strain SSPD1601 share 99% coverage and 100% amino acid identity with the ferric iron uptake transcriptional regulator (Fur protein) of *Vibrio rotiferianus* (WP_010446797.1). In previous works, the *Fur* gene was shown to be involved in iron uptake and the expression of virulence factors [30, 31].

### 3.5. Phylogenetic Analyses

**Figure 3:**
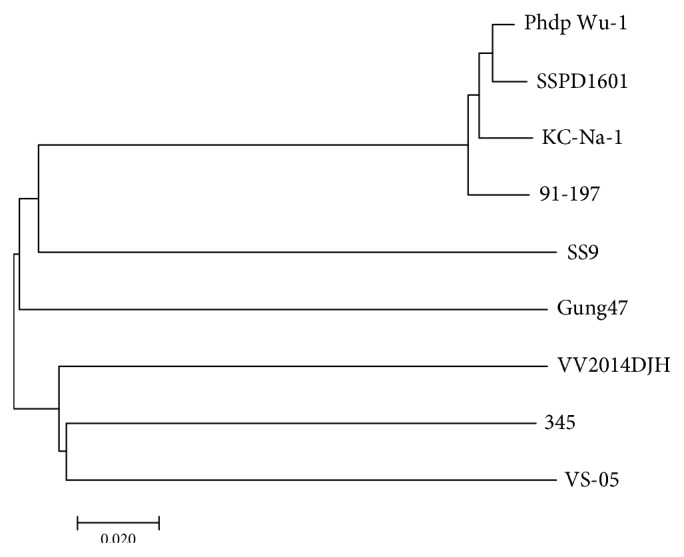
Phylogenetic tree analyses. Phylogenetic tree analysis based on ANI values of the complete genome sequences of PDD KC-Na-1, PDD Phdp Wu-1, *P. damselae* subsp. *piscicida* 91-197, *P. profundum* SS9, *P. gaetbulicola* Gung47, *V. vulnificus* VV2014DJH, *V. harveyi* 345, and *V. scophthalmi* VS-05.

In phylogenetic tree analysis based on ANI values of the selected reference genome sequences, the PDD strain SSPD1601 was most closely related to Phdp Wu-1 (Figure 3). Based on the NCBI description, the Phdp Wu-1 strain was isolated from demersal turbot heart blood in He Bei, China, which is also in the Huang-Bohai Sea area, but the characteristics of the strain have not been described in detail. The results further indicated that different kinds of PDD with strong pathogenicity are widespread in the Huang-Bohai Sea area, with a potential threat to different kinds of marine animals.

### 3.6. Antibiotic Resistance Analyses

By searching ARDB (ardbAnno1.0), only the antimicrobial resistance gene *bacA* was detected in strain SSPD1601, which correlated with resistance to bacitracin. The function of *bacA* was previously described in *Escherichia coli* and *Bacillus*; the gene encodes an undecaprenyl pyrophosphate phosphatase and influences bacitracin biosynthesis [32, 33]. A previous study indicated that the bacitracin resistance gene *bacA* carried by *Bacillus* contributed to its resistance to ultraviolet radiation [34]. The antibiotic resistance phenotype further indicated that PDD strain SSPD1601 was not sensitive to bacitracin. Our results also showed that strain SSPD1601 was resistant to ceftazidime, pipemidic, streptomycin, cefalexin, cefoperazone sodium, acetylspiramycin, clarithromycin, amikacin, gentamycin, kanamycin, oxacillin, ampicillin, and trimethoprim-sulfamethoxazole (Table 1), but the corresponding drug resistance genes were not detected; this could be explained by the lack of targets in SSPD1601 for those antibiotics.

## 4. Conclusions

In summary, the pathogenic and genomic characteristics of SSPD1601 associated with septic skin ulcer in deep-sea cage-cultured black rockfish *S. schlegelii* were revealed. Transmission electron microscopy showed that SSPD1601 is a rod-shaped cell approximately 0.5-0.8 *μ*m × 0.8-1.5 *μ*m with a single polar flagellum. The genome of SSPD1601 includes two chromosomes and two plasmids. The phylogenetic analysis based on ANI indicated that SSPD1601 is most closely related to Phdp Wu-1. Furthermore, the virulence genes *hlyA_ch_* and *hly_ch_* were detected, and three putative hemolysin-encoding genes (*emrA*, *yoaF*, and *VPA0226*) were identified. Additionally, several predicted genes homologous to genes encoding virulence factors flagellin, adhesion enhancer, and OMPs and genes responsible for iron uptake and secretion systems were detected in the genome of SSPD1601. Moreover, the antibiotic resistance phenotype indicated that SSPD1601 is a multidrug-resistant bacterium, but only *bacA* was detected. In summary, our results provide important insight into the genomics of the *Photobacterium* species, promoting a better understanding of the versatility of this genus. Further functional studies will provide new information for the understanding of the pathogenic potential of the organism and prevention of PDD disease.

## Figures and Tables

**Table 1:** Antibiotic resistance phenotype analyses of PDD SSPD1601.

Drugs	Concentration (*μ*g/disc)	Diameter of inhibition (mm)	Sensitivity
Neomycin	30	21	S
Ceftazidime	30	10	R
Pipemidic	30	19	R
Tetracycline	30	20	S
Bacitracin	0.04 U	0	R
Ofloxacin	5	22	S
Streptomycin	10	12	R
Norfloxacin	10	22	S
Cefalexin	9	13	R
Cefradine	30	17	I
Cefoperazone sodium	75	11	R
Cefazolin	30	16	I
Fleroxacin	5	22	S
Acetylspiramycin	30	0	R
Clarithromycin	15	11	R
Amikacin	30	14	R
Gentamycin	10	13	R
Azithromycin	15	14	I
Kanamycin	30	10	R
Oxacillin	1	0	R
Ampicillin	10	0	R
Polymyxin B	300 U	10	I
Florfenicol	30	22	S
Trimethoprim-sulfamethoxazole	25	17	R
Nalidixic acid	30	24	S

Note: S, sensitivity; I, moderate sensitivity; R, resistance.

## Data Availability

The genome sequence of *Photobacterium damselae* subsp. *damselae* strain SSPD1601 has been deposited in the NCBI GenBank server under the SRA accession number PRJNA490082 for chromosome 1, chromosome 2, and the plasmids.

## Supplementary Materials

Supplementary Materials Table S1: virulence factors identified in the genome of PDD strain SSPD1601 according to the Virulence Factors of Bacterial Pathogens database (VFDB).
